# A case of novel, rapidly-growing *Mycolicibacter kumamotonensis* infection in a patient with severe pulmonary disease treated in New York City

**DOI:** 10.1186/s12879-022-07959-2

**Published:** 2023-01-13

**Authors:** Maxwell D. Weidmann, Yuexiu Wu, Fann Wu, Dhrupa D. Hapani, Daniel A. Green, Justin G. Aaron, Gregory J. Berry

**Affiliations:** 1 Department of Pathology and Cell Biology, Columbia University Irving Medical Center, 3959 Broadway, CHC 3-324, New York, NY 10032 USA; 2 Division of Infectious Diseases, Department of Medicine, Columbia University Irving Medical Center, New York, NY USA

**Keywords:** Case report, Non-tuberculous mycobacteria, Kumamotonensis, Post-transplant, Rapid growth

## Abstract

**Introduction:**

*Mycolicibacter kumamotonensis* is a slowly growing, non-chromogenic non-tuberculous mycobacteria (NTM) that was initially distinguished from the *M. terrae* complex in 2006. Since then it has been rarely reported as the cause of pulmonary and soft-tissue infections in both immunocompromised and immunocompetent patients.

**Case presentation:**

We present a case of severe pulmonary disease due to *Mycolicibacter kumamotonensis* in a 57-year-old male who was immunocompetent at time of diagnosis, with a history of interstitial lung disease and a prior diagnosis of tuberculosis (TB). After initial treatment for TB in 2017, his condition stabilized until a recurrence in September 2021, leading to an evaluation for lung transplant in the setting of pulmonary fibrosis and emphysema which led to the identification of *Mycolicibacter kumamotonensis*. A lung transplant was completed, and the patient was successfully treated with a combination of Ethambutol, Azithromycin, and Rifabutin.

**Conclusions:**

This represents the first case reported of *M. kumamotonensis* in a patient undergoing lung transplant, and the first case with rapid culture growth during identification of the organism (4 days). This report highlights the need for consideration of *M. kumamotonensis* as a pathogen in humans, with the potential for rapid growth in liquid media, and the importance of early identification to inform empiric therapy.

## Background

The genus *Mycobacterium* contains a diverse array of species. Those that cause human disease have traditionally been divided into tuberculosis-causing and non-tuberculous mycobacteria (NTM). NTM can be further classified by their rate of growth into rapidly-growing mycobacteria (RGM) and slowly-growing mycobacteria (SGM), depending on whether growth of mature colonies is observed on solid media within 7 days. RGM has most recently been described as containing opportunistic pathogens *M. chelonae-abscessus* complex, *M. smegmatis* group, *M. mucogenicum* group, *M. mageritsense/M. wolinskyi* group*, M. fortuitum* group, and pigmented RGM [3], with the *M. abscessus* complex of particular concern due to its increasing incidence as a cause of opportunistic respiratory, skin and mucosal infections [9].

*Mycolicibacter kumamotonensis*, formerly *Mycobacterium kumamotonense*, is a slowly growing, non-chromogenic NTM that was first distinguished by molecular analysis from the saprophytic *M. terrae* complex in 2006, when initially identified in Japan from a clinical isolate [15]. Since then, reports of pathogenic infections with *M. kumamotonensis* have been sparse, with two cases of pulmonary infection in immunocompetent patients, one of whom had a prior history of TB infection [11, 14] as well as soft-tissue infections such as tenosynovitis of the hand [8]. Considering the differences in management between TB and NTM, awareness of such clinically significant NTM species is important when considering patients with a presumptive history of TB where the identification technique is unclear.

Here we present the first case of rapidly growing NTM identified as *M. kumamotonensis* by both MALDI-TOF and DNA sequencing, in an initially immunocompetent patient with a history of TB and combined pulmonary fibrosis and emphysema (CPFE), who subsequently underwent lung transplant. The complexities of this case demonstrate the need for additional characterization of this emerging NTM species.

## Case presentation

The patient is a 57-year-old male Chinese immigrant with combined pulmonary fibrosis and emphysema and mild pulmonary hypertension, gastroesophageal-reflex disease (GERD), and a prior history of treated TB infection (2016) who presented with a progressive productive cough for three days associated with shortness of breath. The patient was initially admitted to an outside hospital for chronic obstructive pulmonary disease (COPD) exacerbation, but was transferred to our hospital for further management and an expedited lung transplant evaluation (Table 1).

**Table 1:** Timeline of case

	Location	Event	Laboratory findings
Month, Year			
2016	Unknown	Diagnosed at OSH with emphysema, pulmonary fibrosis	
2017	China	Diagnosed with TB in China, treatment on return managed by NYS DOH	
2018	NYP Queens	Started on Nintedanib for pulmonary fibrosis, cavitary lesion first demonstrated on Chest CT	
May, 2021	NYP CUMC	Initiated lung transplant evaluation	
Hospital Day			
− 22	NYP Queens	Initial complaint of fever, chills and right upper chest pain	
− 19		Admitted for presumed COPD exacerbation	
− 18			Respiratory culture positive for *H. parainfluenzae*
− 6		Completes 12 day course of CTX with symptomatic improvement	
1 (Sept 2021)	NYP CUMC	Transferred for lung transplant evaluation with ongoing elevated O2 requirement	
8	NYP CUMC MICU	Transferred to ICU with increased SOB	
9		Placed on VA-ECMO, initial AFB culture collected (tracheal aspirate)	
10			BAL AFB smear negative
14			2nd BAL AFB smear positive, MTB/Rif NAAT negative. 1st BAL sample liquid culture flags positive for AFB (4-days growth)
16	OR	Double lung transplant performed, discharged to CTICU, started on empiric NTM therapy (azithro, mero and amikacin)	
22	NYP CUMC CTICU	Therapy changed to azithromycin, ethambutol and amikacin	Identification of *M. kumamotonensis* by MALDI-TOF from initial AFB specimen (collected HD 9),
30			ID of initial BAL specimen confirmed by sequencing (hsp65)
32			Collection date of first specimen for AFB culture resulting as negative
87	NYP CUMC Outpatient		Antimicrobial susceptibilities returned, amikacin changed to rifabutin
180		Concludes therapy with all AFB monitoring negative	

**Fig. 1:**
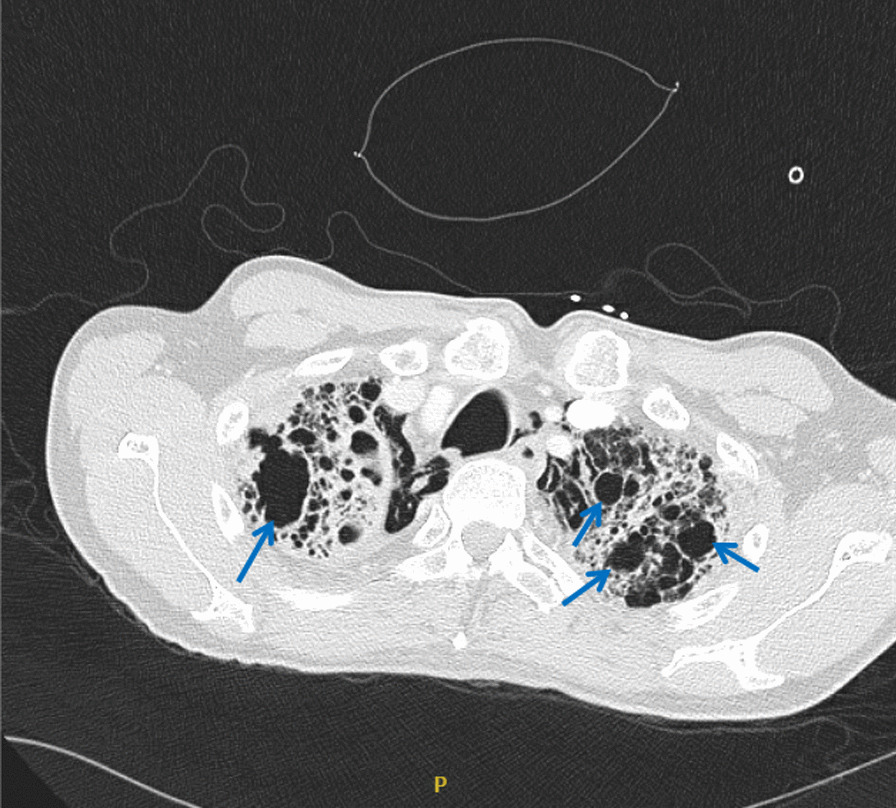
Cavitary lesions in a patient with *M. kumamotonensis* infection and underlying pulmonary fibrosis. Chest CT showed bilateral upper lobe cavitary lesions (see arrows) and honeycombing. Cavities in the right apex are more prominent than left apex

In 2016, the patient initially developed a progressive productive cough and shortness of breath and was diagnosed with emphysema and pulmonary fibrosis. In 2017, while visiting China, he was hospitalized for 10 days and diagnosed with tuberculosis, (though methods of diagnosis were not known to the patient, or available in the medical record) and was subsequently treated with five unidentified oral medications for 6 months. Subsequently, the patient had continued stable shortness of breath on exertion, necessitating three liters of home oxygen. He was started on Nintedanib in 2018, and in 2020 chest computed tomography (CT) imaging demonstrated progressive changes with a right upper cavitary lesion that was retrospectively determined to have originated in 2018 (Fig. 1). In spring 2021, he was referred for an outpatient lung transplant evaluation. Of note, an Interferon-Gamma Release Assay (IRGA) was performed during this evaluation, prior to the current admission, and resulted as positive.

The patient’s pulmonary symptoms remained relatively stable until September 2021, when he presented with worsening dyspnea and cough productive of green sputum that progressed during the 3 days prior to admission. He also reported a fever and chills 3 days prior to admission with right upper chest pain. He was admitted for a presumed COPD exacerbation with oxygen (O_2_) requirements increasing to maximum non-invasive settings (12–15 L on Venti-mask with FiO2 of 50–100%) for which he was treated with albuterol/ipratropium as well as an oral prednisone taper over a two-week period and maintained on a 10 mg dosage. His initial laboratory results showed no leukocytosis, a normal procalcitonin level, a negative troponin test, and normal brain natriuretic peptide (BNP) level.

Respiratory cultures grew *Haemophilus parainfluenzae* within 4 days of admission and he was treated with a 12-day course of intravenous ceftriaxone (Table 1). There was subsequent improvement in his symptoms, but his oxygen requirement was still elevated from baseline (8-10L at FiO_2_ of 50% on Venti-mask), and he was transferred to our hospital for further transplant evaluation (hospital day 1, HD1). Chest imaging showed upper lobe bronchiectasis greater on the right side and cavitary changes concerning for possible fungal cavitation. He also had a 1,3-*beta-*d-glucan level > 500 pg/ml, for which he was started empirically on voriconazole on HD10, but fungal cultures were consistently negative throughout his hospital course.

He developed increasing shortness of breath (SOB) on HD8, and was transferred to the intensive care unit, where he was found to have worsening pulmonary hypertension and was placed on extracorporeal membrane oxygenation (venoarterial (VA)-ECMO), with antibiotics broadened to piperacillin-tazobactam and vancomycin. A double lung transplantation was performed on HD16. His post-transplant course was complicated by cardiogenic shock, acute kidney injury, pneumothorax, and pulmonary edema. He was decannulated from VA-ECMO on HD19 and extubated on HD23.

His extensive microbiologic workup included a tracheal aspirate specimen sent for acid-fast bacilli (AFB) culture on HD9, prior to transplant, with an auramine-rhodamine stain performed that resulted as negative. A bronchoalveolar lavage (BAL) specimen from HD10 was also stain-negative, but a second BAL specimen collected on HD14 was found to be positive for AFB by auramine-rhodamine staining. Nucleic acid amplification testing from the positive BAL specimen was negative for *M. tuberculosis* (MTB) complex (Xpert MTB/Rif, Cepheid, Sunnyvale, CA), and empiric therapy for NTM was initiated with azithromycin, meropenem, and amikacin on the date of transplant while awaiting species identification. Minocycline was also initiated for a positive BAL culture for *Stenotrophomonas maltophilia.* AFB and bacterial cultures from the OR at the time of transplant were positive for rare AFB and grew *Acinetobacter baumannii* from the donor lung BAL bacterial culture. Growth was detected in broth culture (BACTEC MGIT 960 system with modified Middlebrook 7H9 broth, BD Biosciences, Sparks, MD) from the initial BAL specimen (HD10 to HD14) but there was insufficient biomass for identification by Matrix-Assisted Laser Desorption Ionization-Time of Flight Mass Spectrometry (MALDI-TOF MS, Bruker Daltonics, Billerica, MA). The liquid broth culture from the initial tracheal aspirate specimen (HD9) flagged positive for a micro-precipitate and was subcultured onto Middlebrook 7H10 medium on HD16 and was found to be positive on HD22 with MALDI-TOF identification as *M. kumamotonensis* using the Bruker Mycobacteria database.

**Fig. 2:**
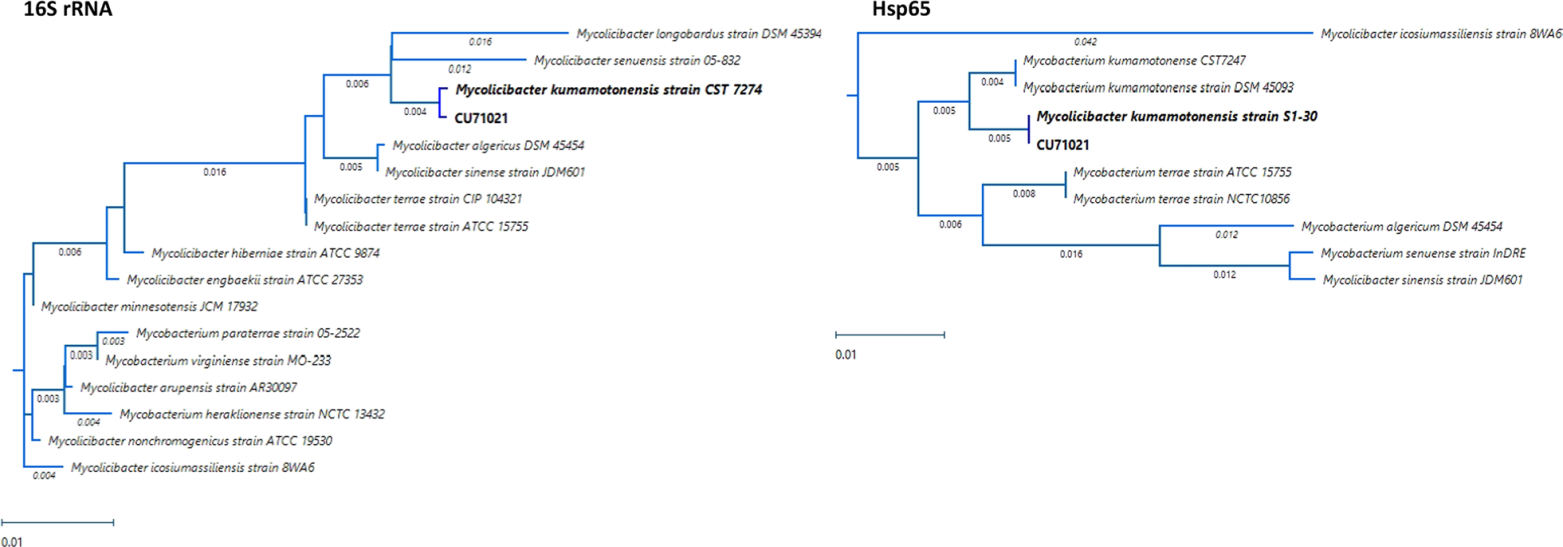
Phylogenetic trees for the members of *Mycobacterium terrae* complex, based on 16S rRNA (1487 bp) and hsp65 genes (441 bp). The sequence of 16S rRNA and hsp65 genes show 99.5% (1421/1424 identities) and 99.1% similarity (420/424 identities) compared with reported sequences of *M. kumamotonensis* (strain CST7274) [15]. hsp65 gene shows 100% (409/409 identities) match with *M. kumamotonensis* S1-30

The initial BAL specimen (HD10) was confirmed as *M. kumamotonensis* by *hsp65* gene amplification and sequencing of the 441 base-pair fragment with alignment to known sequences in GenBank (https://www.ncbi.nlm.nih.gov/genbank/) on HD30 and 16S rRNA gene amplification was also performed and aligned to known sequences in GenBank for further confirmation (Fig. 2). Phylogenetic trees were generated from alignment results using Lasergene MegAlign Pro software from DNASTAR, Inc. (DNASTAR, Madison, WI USA). Antimicrobial susceptibility testing was performed at National Jewish Medical Center (Denver, CO), with the following results: Rifabutin (S), Moxifloxacin (R), Amikacin (I), Linezolid (R), Ciprofloxacin (R), Streptomycin (NI), Clarithromycin (S), Rifampin (R), Ethambutol (NI); Rif + Etham (NE), Rifampin combo (TI), Ethambutol combo (TS); Doxycycline (R), Trimethoprim-sulbactam (S), Clofazimine (NI), Minocycline (R) (S = susceptible, R = resistant, NI = no interpretation, NE = no effect, TI = tentative intermediate, TS = tentative susceptible). The patient’s initial therapy was oral azithromycin, ethambutol and intravenous amikacin based on the organism identification. Once susceptibility results were available, therapy was modified to ethambutol, azithromycin, and rifabutin for 6 months from first negative AFB culture. The patient continued to tolerate the regimen well with no adverse effects, aside from possible renal toxicity detected via changes in creatinine clearance, which was addressed by subsequent reductions in the weekly ethambutol dosage. The patient underwent dose adjustment of the tacrolimus used in his immunosuppression regimen once initiated on rifabutin.

Subsequent AFB cultures collected on HD15, HD18 and HD21 (post-transplant) were also found to be positive for *M. kumamotonensis* by MALDI-TOF. Subsequent AFB specimens from HD32, HD38, HD45, HD58 and HD73 were found to be negative for AFB.

## Discussion and conclusions

In this case report, we describe the first case of rapidly-growing *Mycolicibacter kumamotonensis*, an NTM that has usually been described as slowly growing. Additionally, we report the first such identification in a clinical isolate in the United States, as well as the first case in which the patient subsequently underwent lung transplantation.

### Rapidly-growing *M. kumamotonensis*

Rapidly-growing *Mycobacteria* (RGM) include six major taxonomic groups that grow mature colonies on solid media within 7 days (Brown-Elliott and Philley, 2017). Pulmonary disease from RGM is most commonly due to *M. abscessus,* which is especially concerning given the associated morbidity for lung transplant recipients (Brown-Elliott and Philley, 2017). In this case, an infectious diseases specialist was consulted on the day of transplant surgery when a RGM was first identified in culture. Extensive evaluation was performed prior to surgery, at which time AFB smears from tracheal aspirate and BAL specimens were both negative, but growth of NTM was observed in culture. While previous clinical reports of *M. kumamotonensis* have documented culture incubation times of more than 2 weeks [11, 22], the isolates in this case were recovered within 4–6 days of culture incubation in a patient with severe pulmonary infection.

*Mycolicibacter kumamotonensis* is a relatively rare *Mycobacterium* species with only a few clinical case reports and no detailed laboratory studies describing culture incubation times. In contrast, TB is frequently identified in clinical practice and studies have shown that the time it takes to recover in culture has strong negative correlation with bacterial burden [1]. Shorter recovery times are also associated with a higher risk of transmission and relapse after therapy [2, 17]. Moreover, it was recently reported that cavitary pulmonary TB has significantly shorter culture incubation time than non-cavitary TB or extra-pulmonary TB [24]. Given the known associations with culture incubation time for TB, the short culture incubation time for *M. kumamotonensis* in this case implies a high bacterial load, which might have contributed to the severity of lung disease in this case. The patient also grew other organisms as discussed above, which may have also contributed to his worsened underlying pulmonary fibrosis. Further studies are needed to assess the relationship between culture incubation time and disease severity for *M. kumamotonensis* pulmonary infection.

### *Mycolicibacter kumamotonensis* infection in a patient with a history of tuberculosis

Misidentification of *M. kumamotonensis* as TB has been reported in pulmonary and extra-pulmonary infections [11, 14, 18, 22], which was attributed to similar clinical manifestations and cross-reactivity of commercial molecular probes, factors that have also been associated with misdiagnosis of a wide variety of other NTM as TB [4, 12]. Previous studies have also shown identification of *M. kumamotonensis* in clinical samples from patients with suspected tuberculosis [7, 10, 13]. Hoza et al. [7] also showed that 1.3% of individuals had both *M. tuberculosis* and NTM, including *M. kumamotonensis*. Co-infection with NTM and TB is uncommon, but not rare [19]. A recent publication documented co-infection with TB and three species of NTM [25]. Although patients with such co-infections usually manifest symptoms of tuberculosis*,* the presence of an additional NTM may contribute to disease pathogenesis, severity and progression [19].

Patients with underlying lung diseases have a higher incidence of NTM infection. Studies conducted in Asia have demonstrated that 35–37% of NTM patients had a prior history of tuberculosis [10, 21]. It is often uncertain whether the NTM infection is a superimposed secondary infection or was present from prior colonization. The patient we reported here is a Chinese immigrant who had a history of chronic pulmonary fibrosis and emphysema diagnosed in 2016 and pulmonary tuberculosis diagnosed in 2017 during a trip in China. He completed a course of anti-tuberculous therapy upon return to the United States with documented clearance of sputum AFB cultures. However, a thoracic CT chest in 2020 demonstrated progressive changes with a right upper cavitary lesion reported since 2018. The upper lobe cavitary lesions were initially considered bullous changes attributed to his underlying pulmonary fibrosis. However, the subsequent diagnosis of *M. kumamotonensis* infection as described in this case raises the possibility that this organism may have contributed to the progression of his lung disease. Furthermore, administration of steroids without anti-mycobacterial coverage may have contributed to accelerated disease progression. The laboratory workup of TB in 2017 is also unknown, and it is therefore possible that *M. kumamotonensis* was misidentified as TB or that the patient had co-infection with TB and *M. kumamotonensis* at that time. *M. kumamotonensi*s may be resistant to some agents included in standard anti-tuberculous therapy (such as rifampin) and inadequate treatment may cause relapse over time [14]. A new superimposed *M. kumamotonensis* infection is also possible, but given the progressive clinical and radiographic changes following TB and the geographic distribution of *M. kumamotonensis,* the findings suggest that this organism had contributed to progressive pulmonary infection over a longer period of time.

### First reported *M. kumamotonensis* infection in the US

The geographic diversity of pulmonary NTM has been analyzed worldwide [6], and previous authors have characterized the distribution of *M. kumamotonensis* across multiple countries and regions [5, 16]. Isolation of *M. kumamotonensis* has been reported in Japan [8, 15], Mexico 22, Greece [11, 14], China 13, Tanzania [7], Zambia [16], Korea [10], Turkey [5], Italy 23, and Spain [18]. This case is the first reported in the US.

### First reported *M. kumamotonensis* infection in lung transplant recipient

Lung transplant recipients are at higher risk of NTM infection than the general population. Shah et al. showed that 14% of lung recipients developed NTM respiratory tract infections post lung transplantation, most commonly with *M. abscessus* and *M. avium* complex [20]. The same study revealed a significant increase in the rate of bronchiolitis obliterans syndrome in patients with NTM infection. In this case, a NTM was initially recovered on the date of transplant surgery, and appropriate empiric therapy was initiated immediately after surgery. The initial treatment course consisted of amikacin, azithromycin and meropenem. Ethambutol was added upon speciation, amikacin was discontinued and rifabutin was initiated based on the expected resistance profile of *M. kumamotonensis*. Subsequent AFB smears were negative after 2 days of treatment, subsequent AFB cultures were negative after 2 weeks of therapy, and the patient completed 6 months of therapy after first negative AFB culture with no clinical signs of disease on follow-up. Although the optimal treatment regimen for this organism is not clear given its rarity, the decision to simplify therapy to a three-drug regimen and stop therapy was made based on the relatively rapid conversion to negative AFB cultures. It is likely that this rapid conversion was also significantly aided by the substantial decrease in microbial burden occurring from the explant of the patient’s diseased lungs at the time of double lung transplantation.

In summary, this case demonstrates the need for increased awareness of *M. kumamotonensis* as a potential human pathogen, especially in patients with history of TB or underlying pulmonary disease. With the implementation of newer identification methods like MALDI-TOF MS and genetic sequencing, there may be an increase in reported cases of this rare NTM. As *M. kumamotonensis* has a different susceptibility profile than TB, early identification is critical to empiric therapy decisions, and antimicrobial susceptibility testing should be performed to guide definitive therapy.

## Data Availability

The 16S ribosomal RNA sequence data generated and analyzed during the current study are available in the GenBank repository, OP809655. The Hsp65 sequence data generated and analyzed during the current study are available in the GenBank repository, OP820520.

## Abbreviations

NTM: Non-tuberculous mycobacteria
TB: Tuberculosis
RGM: Rapidly-growing mycobacteria
SGM: Slowly growing mycobacteria
CPFE: Combined pulmonary fibrosis and emphysema
GERD: Gastro-esophageal reflux disease
COPD: Chronic obstructive pulmonary disease
CT: Computed tomography
HD: Hospital day
O_2_: Oxygen
BNP: Brain natriuretic peptide
SOB: Shortness of breath
VA-ECMO: Venoarterial extracorporeal membrane oxygenation
AFB: Acid-fast bacilli
BAL: Bronchoalveolar lavage
MALDI-TOF: Matrix assisted laser desorption/ionization-time of flight

## References

[1] Bark CM, Okwera A, Joloba ML, Thiel BA, Nakibali JG, Debanne SM, Boom WH, Eisenach KD, Johnson JL (2011). Time to detection of *Mycobacterium tuberculosis* as an alternative to quantitative cultures. Tuberculosis (Edinb).

[2] Bark CM, van Thiel BA, HsuJohnsoneh JL (2013). Pretreatment time to detection of Mycobacterium tuberculosis in liquid culture is associated with relapse after therapy. J Clin Microbiol.

[3] Brown-Elliott BA, Philley JV (2017). Rapidly growing mycobacteria. Microbiol Spectr..

[4] Butler WR, O’Connor SP, Yakrus MA, Gross WM (1994). Cross-reactivity of genetic probe for detection of *Mycobacterium tuberculosis* with newly described species *Mycobacterium celatum*. J Clin Microbiol.

[5] Gunaydin M, Yanik K, Eroglu C, Sanic A, Ceyhan I, Erturan Z, Durmaz R (2013). Distribution of nontuberculous *Mycobacteria strains*. Ann Clin Microbiol Antimicrob.

[6] Hoefsloot W, van Ingen J, Andrejak C, Angeby K, Bauriaud R, Bemer P, Beylis N, Boeree MJ, Cacho J, Chihota V, Chimara E, Churchyard G, Cias R, Daza R, Daley CL, Dekhuijzen PN, Domingo D, Drobniewski F, Esteban J, Fauville-Dufaux M, Folkvardsen DB, Gibbons N, Gómez-Mampaso E, Gonzalez R, Hoffmann H, Hsueh PR, Indra A, Jagielski T, Jamieson F, Jankovic M, Jong E, Keane J, Koh WJ, Lange B, Leao S, Macedo R, Mannsåker T, Marras TK, Maugein J, Milburn HJ, Mlinkó T, Morcillo N, Morimoto K, Papaventsis D, Palenque E, Paez-Peña M, Piersimoni C, Polanová M, Rastogi N, Richter E, Ruiz-Serrano MJ, Silva A, da Silva MP, Simsek H, van Soolingen D, Szabó N, Thomson R, Tórtola Fernandez T, Tortoli E, Totten SE, Tyrrell G, Vasankari T, Villar M, Walkiewicz R, Winthrop KL, Wagner D, Nontuberculous Mycobacteria Network European Trials Group (2013). The geographic diversity of nontuberculous mycobacteria isolated from pulmonary samples: an NTM-NET collaborative study. Eur Respir J.

[7] Hoza AS, Mfinanga SG, Moser I, König B (2016). Molecular characterization of *Mycobacterium tuberculosis* isolates from Tanga, Tanzania: first insight of MIRU-VNTR and microarray-based spoligotyping in a high burden country. Tuberculosis (Edinb).

[8] Iemura-Kashiwagi M, Ito I, Ikeguchi R, Kadoya M, Iemura T, Yoshida S, Suzuki K, Hirai T (2020). Soft tissue infection caused by *Mycolicibacter kumamotonensis*. J Infect Chemother.

[9] Johansen MD, Herrmann JL, Kremer L (2020). Non-tuberculous mycobacteria and the rise of *Mycobacterium abscessus*. Nat Rev Microbiol.

[10] Kim J, Seong MW, Kim EC (2015). Frequency and clinical implications of the isolation of rare nontuberculous mycobacteria. BMC Infect Dis.

[11] Kontos F, Mavromanolakis DN, Zande MC, Gitti ZG (2016). Isolation of *Mycobacterium kumamotonense* from a patient with pulmonary infection and latent tuberculosis. Indian J Med Microbiol.

[12] Lefmann M, Moter A, Schweickert B, Göbel UB (2005). Misidentification of *Mycobacterium leprae* as Mycobacterium intracellulare by the COBAS AMPLICOR *M. intracellulare* test. J Clin Microbiol.

[13] Liu H, Lian L, Jiang Y, Huang M, Tan Y, Zhao X, Zhang J, Yu Q, Liu J, Dong H, Lu B, Wu Y, Wan K (2016). Identification of species of nontuberculous mycobacteria clinical isolates from 8 provinces of China. Biomed Res Int.

[14] Manika K, Kontos F, Papavasileiou A, Papaventsis D, Sionidou M, Kioumis I (2021). Severe pulmonary disease caused by *Mycolicibacter kumamotonensis*. Emerg Infect Dis.

[15] Masaki T, Ohkusu K, Hata H, Fujiwara N, Iihara H, Yamada-Noda M, Nhung PH, Hayashi M, Asano Y, Kawamura Y, Ezaki T (2006). *Mycobacterium kumamotonense* Sp. Nov. recovered from clinical specimen and the first isolation report of *Mycobacterium arupense* in Japan: novel slowly growing, nonchromogenic clinical isolates related to *Mycobacterium terrae* complex. Microbiol Immunol.

[16] Mwikuma G, Kwenda G, Hang'ombe BM, Simulundu E, Kaile T, Nzala S, Siziya S, Suzuki Y (2015). Molecular identification of non-tuberculous mycobacteria isolated from clinical specimens in Zambia. Ann Clin Microbiol Antimicrob.

[17] O'Shea MK, Koh GC, Munang M, Smith G, Banerjee A, Dedicoat M (2014). Time-to-detection in culture predicts risk of *Mycobacterium tuberculosis* transmission: a cohort study. Clin Infect Dis.

[18] Rodríguez-Aranda A, Jimenez MS, Yubero J, Chaves F, Rubio-Garcia R, Palenque E, García MJ, Menendez MC (2010). Misindentification of *Mycobacterium kumamotonense* as *M. tuberculosis*. Emerg Infect Dis.

[19] Sarro YD, Kone B, Diarra B (2018). Simultaneous diagnosis of tuberculous and non-tuberculous mycobacterial diseases: time for a better patient management. Clin Microbiol Infect Dis.

[20] Shah SK, McAnally KJ, Seoane L, Lombard GA, LaPlace SG, Lick S, Dhillon GS, Valentine VG (2016). Analysis of pulmonary non-tuberculous mycobacterial infections after lung transplantation. Transpl Infect Dis.

[21] Simons S, van Ingen J, Hsueh PR (2013). Nontuberculous mycobacteria in respiratory tract infections, eastern Asia. Emerg Infect Dis.

[22] Solis AH, González-Villa M, Ramírez-González JE, Colin-Muñoz Y, Cicero-Sabido R (2019). *Mycobacterium kumamotonense* in the cervical region in an immunocompetent patient, clinical case report in Mexico. J Infect Dev Ctries.

[23] Tortoli E, Gitti Z, Klenk HP, Lauria S, Mannino R, Mantegani P, Mariottini A, Neonakis I (2013). Survey of 150 strains belonging to the *Mycobacterium terrae* complex and description of *Mycobacterium engbaekii* sp. nov., *Mycobacterium heraklionense* sp. nov. and *Mycobacterium longobardum* sp. nov. Int J Syst Evol Microbiol.

[24] Vongthilath-Moeung R, Poncet A, Renzi G, Schrenzel J, Janssens JP (2021). Time to detection of growth for *Mycobacterium tuberculosis* in a low incidence area. Front Cell Infect Microbiol.

[25] Wang L, Liu D, Yung L, Rodriguez GD, Prasad N, Segal-Maurer S, Singh V, Vikram E, Zou A, Cheng G, Rodgers WH (2021). Co-infection with 4 species of mycobacteria identified by using next-generation sequencing. Emerg Infect Dis.

